# E-learning is a burden for the deaf and hard of hearing

**DOI:** 10.1038/s41598-022-13542-1

**Published:** 2022-06-04

**Authors:** Filipa M. Rodrigues, Ana Maria Abreu, Ingela Holmström, Ana Mineiro

**Affiliations:** 1 Universidade Católica Portuguesa, Institute of Health Sciences, Center for Interdisciplinary Research in Health, Lisbon, Portugal; 2 School of Education and Social Sciences, Polytechnic of Leiria, Leiria, Portugal; 3 Department of Linguistics, Stockholm University, Stockholm, Sweden

**Keywords:** Human behaviour, Psychology, Environmental social sciences

## Abstract

When considering deaf and hard of hearing (DHH) population, research recognizes that fatigue due to communication challenges and multi-focal attention allocation is a significant concern. Given the putative heightened demands of distance learning on deaf and hard of hearing students, we investigate how an online environment might differently affect deaf and hard of hearing participants, compared to hearing participants, Portuguese Sign Language (PSL) users and non-users. Our findings show that the deaf and hard of hearing group present higher values in the post-task fatigue rates with significant differences from the hearing group (non-PSL users). Furthermore, our results revealed an association between post-task fatigue rates and lower performance scores for the deaf and hard of hearing group, and the gap is significantly bigger when compared with the hearing group (non-PSL users). We also found evidence for high levels of post-task fatigue and lower performance scores in the hearing group PSL users. These novel data contribute to the discussion concerning of the pros and cons of digital migration and help redesign more accessible and equitable methodologies and approaches, especially in the DHH educational field, ultimately supporting policymakers in redefining optimal learning strategies.

## Introduction

Despite the growing investment in the study of the relationship between hearing loss and fatigue, the available research does not demonstrate, as clearly as would be expected, the causal relationship between levels of effort and subsequent fatigue in deaf and hard of hearing individuals^1^. Research has not yet found an irrefutable hypothesis that relates hearing loss, effort (cognitive effort and physical effort), and higher fatigue levels (typology and duration) in DHH participants, than in those of hearing participants, when studying the comparison between samples, as other idiosyncratic variables seem to have a significant impact in self-reported fatigue levels^2^. Considering that the concept of fatigue is a complex concept to study, the choice of data collection testing methods also proves to be a challenge^3,4^, especially when studying such a heterogeneous population. The bibliographical research demonstrates that there is still room for further investigation in the area, since the impact of fatigue dimensions in DHH adults’ lives is still little known. Nevertheless, literature maintains that the additional attention, concentration, and effort needed to overcome the communicative problems associated with hearing loss, in hearing aid users especially, result in increased reports of auditory effort, stress and fatigue compared to individuals with normal hearing^5,6^. It has been shown that high levels of effort daily may result in mental fatigue in the DHH population, with associated reduced ability to concentrate or to perform any cognitive task^6^, particularly when potentially interfering distractors are present^7,8^. Despite several non-consensual aspects, the definition of Cognitive Fatigue (CF) is generally considered as a decrease in, or inability to sustain, task performance throughout the duration of a sustained attention task^9^. CF is associated with impaired cognitive control^10^ high-level information processing^11^ and sustained attention^12^. Although widely studied, the phenomenon of fatigue and its negative impact on the quality of life of contemporary societies, the scientific community has made little progress in studying the putative impact of fatigue in DHH youngsters and adults and the resulting constraints that negatively affect the academic, professional, and social dimensions^13^.

Existing research has recognized the increased difficulties of DHH students regarding the lower effectiveness of the instructional material presented in class when there is an overlap of semiotic resources^14^. Research into the multilingual or ‘translingual’ communicative classroom practices has encompassed a focus on multimodality^15^ stressing the need to re-think simultaneous communicative actions in more efficient sequential ‘chaining’ of modes, to avoid semiotic overlap and thus, increased levels of effort and subsequent communication difficulties and fatigue. We find claims, in the literature, that using communication and information technologies (ICT) increases the learning capacities of DHH students, especially when multimedia resources are designed according to their specific needs^16^. The desired effectiveness of using multimedia in teaching DHH students remains, however, dependent on the efficiency of the instructional design that should consider modern learning theories, like the Cognitive Load Theory (CLT). This theory assumes that a cognitive load occurs when cognitive processing requirements exceed the capacities already available to students^17^. Researchers state that the CLT is concerned with the instructional implications of interaction between information structures and cognitive architecture. However, in tandem with the “interactivity” element, the way in which information is presented to learners and the learning activities required can also impose a cognitive load^18^. Exposure to High Cognitive Load (HCL) levels, in conditions where the time to process ongoing cognitive demands is restricted, also leads to increased Cognitive Fatigue^19^. Within the Cognitive Theory of Multimedia Learning (CTML), cognitive load on DHH students can be measured^17^, ^20,21^ in dimensions as mental demand, physical demand, temporal demand, performance, effort, and frustration. Findings showed that the use of multimedia resources proved to be insufficient in the acquisition of scientific concepts by deaf students of elementary / high school, since, if poorly designed, the multimedia presentations can increase the levels of Cognitive Load and act as a barrier to the learning process, instead of acting as a facilitator. Given the quantity and diversity of informational modalities, studies indicate that presentation format may make it difficult for students to grasp the taught concepts effectively^17,20,21^.

As we witness a COVID-19 motivated push towards digital migration with the transition to online work and online classes speeding up without careful impact analyses, the effects of this accelerated transition towards distance learning modalities within the DHH population, must be thoroughly investigated. Here we search for evidence on the putative differential impact that a traditional model of an *e-learning* situation might have on DHH compared to PSL hearing users and non-users^22^.

We believe that due to COVID-19, given the short time to adapt to distance learning scenarios, learning situations migrated rapidly to virtual environments without the necessary adjustments in the design of multimedia resources, specifically for the DHH population, namely, to concern issues of cognitive load and fatigue^23^. Given the putative heightened demands of distance learning on DHH students, we aim to investigate how an online environment might differently affect DHH compared to Hearing participants^24,25^. Here, we infer the consequential fatigue involved in an *e-learning* situation based on performance and fatigue scores. In line with this, we applied a Fatigue Assessment Scale (FAS) to quantify fatigue before the experiment procedure, upon participant recruitment. The FAS is a validated and standardized self-report Likert scale^26–28^. We used it to generate an initial baseline for comparison with later results from this study. Immediately after the *e-learning* presentation, the participants also indicated on a Visual Analogue Scale (VAS)^29–33^, the level of mental and physical fatigue perceived post-task and proceeded to submit a performance test, pertaining to information conveyed in the online class.

## Methods

### Participants

We chose an *ex-post facto* experimental type design for which we developed an ecological *e-learning* situation wherein the information conveyed was kept constant across selected groups, while comparing fatigue and learning outcomes between Portuguese adult samples (n = 51), namely: a group of deaf and hard of hearing participants (DHH; n = 17) proficient in Portuguese sign language, a group of hearing participants (PSL; n = 17) proficient in PSL and a control group of hearing participants unfamiliar with sign language (C; n = 17).

Individuals identified themselves as DHH/hearing individuals, Sign Language proficient users upon recruitment for this study. The procedure was similar for individuals unfamiliar with PSL.

To fulfil the requisites for parametric testing^34^ when dealing with 2 to 9 groups, we strived to recruit more than 15 participants per group. Participants were recruited via convenience sampling. All methods were carried out in accordance with the Declaration of Helsinki^35^ guidelines for human research and approved by the University’s local ethics committee (*Comissão de Ética para a Saúde da Universidade Católica Portuguesa*). All participants gave their informed consent prior to enrolment. The images that directly identify people involved in the study are from one of the researchers (the presenter) and from the Portuguese Sign Language Interpreter, who gave informed consent for publication of identifying images in an online open-access publication.

### Task design and procedure

**Figure 1:**
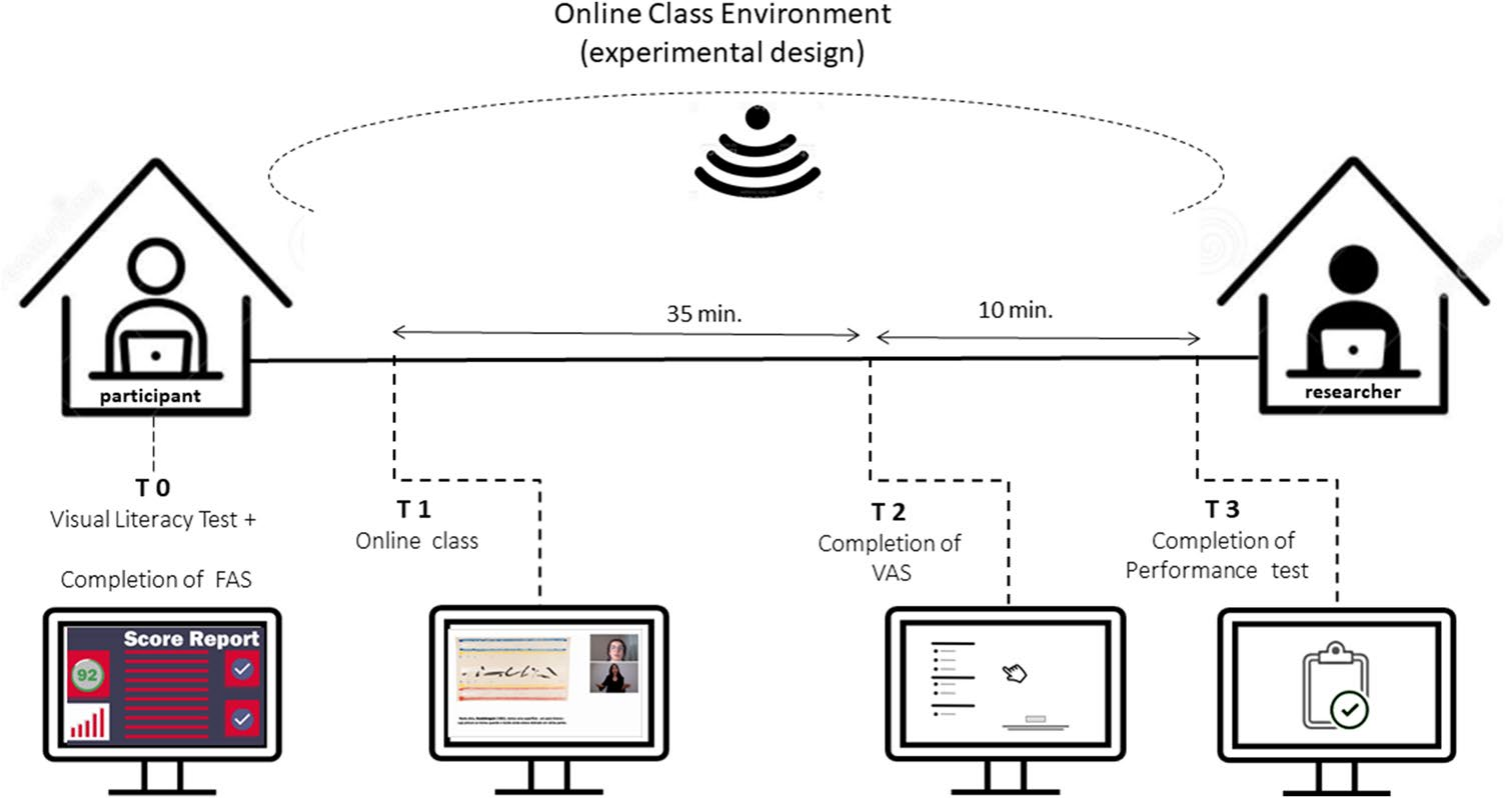
Experimental design (sequence and timeline).

The study was developed across four moments—in T0: completion of an online visual literacy test (in tandem with participant recruitment) and of the Fatigue Assessment Scale (FAS); T1: online class attendance; T2: VAS completion measuring both mental and physical fatigue and T3: performance questionnaire (Fig. 1).

The participants were requested to access and complete online forms, containing the self-completion scales and 2 tests: an art literacy test, the FAS, a VAS to assess mental and physical fatigue levels and a multiple-choice performance Test. During the whole procedure, participants were assessed individually.

### Visual art literacy test

To form homogeneous groups in terms of visual art literacy, participants completed an online test upon recruitment. Visual literacy pertains to the knowledge and use of visual elements in visual communication, knowledge and use of specific vocabulary, and the ability to present, respond, and connect through symbolic and metaphoric forms that are unique to the visual arts. The test consisted of 10 multiple-choice questions with a score of 10 points each, concerning the information conveyed by different sets of images.

### The Fatigue Assessment Scale (FAS)

At the time of recruitment, participants were asked to complete a fatigue rating scale- assessment Fatigue Scale (FAS). The FAS score is obtained from a 10-item scale that evaluates symptoms of chronic fatigue. Some examples of FAS questions are: "Physically I feel exhausted", "I have trouble thinking with clarity" or "Fatigue bothers me" (see Supplementary Information S1). The scale had a filling time of approximately 2–3 min, without a time limit. We used a validated and authorized Portuguese version of FAS^36^. We chose to apply the FAS questionnaire before experimental manipulation to obtain an initial global reading of the sample groups, regarding fatigue reported in a daily basis. The aim was to differentiate the baseline fatigue level for all groups and used it afterwards in the interpretation of later findings from the VAS, the performance test and for the discussion.

### The Videographic stimuli

**Figure 2:**
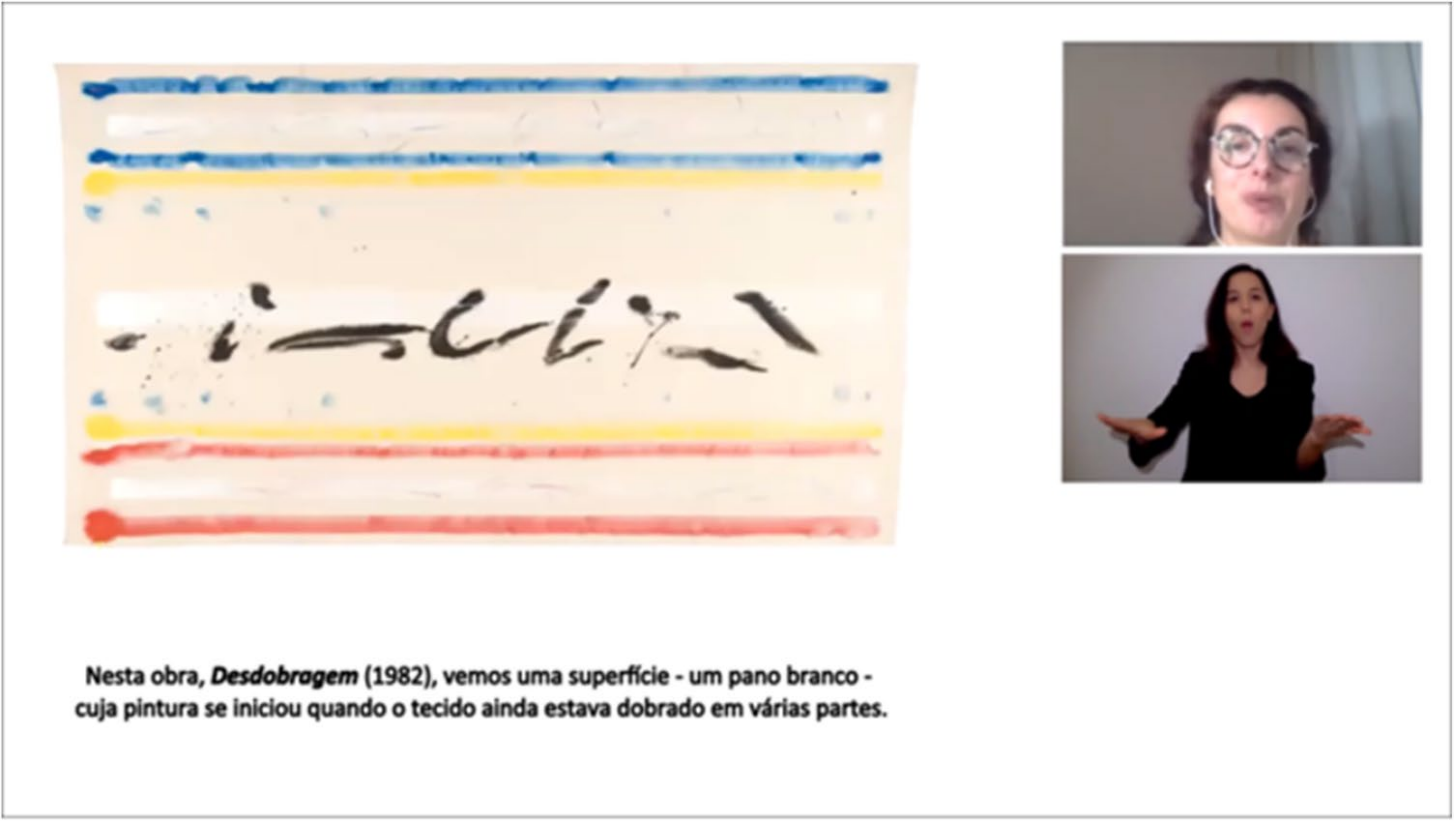
Still frame of the online class presentation designed to mimic a typical online presentation with visual to the left, written information in the bottom and presenter and PSL translator squares to the right.

After the tasks performed upon recruitment, the tasks to be performed following the experimental design were scheduled: viewing the online class followed by filling out the VAS and the performance testing. For this purpose, participants received a new Zoom link to access these contents. The introduction to the online class was presented (voice and image of the presenter) in oral and written (Portuguese Language) in tandem with Sign (Portuguese Sign Language). During this, the presenter gave instructions for the completion of task. The online class content started immediately after this brief information and contemplated information concerning four different works of art. Art works allow the establishment of bridges between the visual-verbal language through the "reading" of the visual narrative of the pictorial composition. We consider, therefore, artistic teaching to be a very relevant topic since it showcases information transmission according to the multimodal dimension. The information conveyed was based on images of works of art and its description, presenting a simultaneous combination of semiotic resources of dual nature (visual and verbal). The presentation lasted 35 min and contained information about artworks, the artists and historical contextualization. To achieve this, we designed a screen display with the simultaneous presentation of information in the following modalities: visual and auditory (teacher) visual (stimulus to be learned), sign language (PSL translator) and written topics corresponding to the presented discourse. For each work, the presenter transmitted oral information for approximately 10 min, with simultaneous translation in Portuguese Sign Language. The written information appeared as a short sentence at the bottom of the screen (type of short caption called "oracles"). Regarding the presentation format, the following screen display structure was presented to all participants, designed to mimic a typical online presentation (Fig. 2).

**Figure 3:**
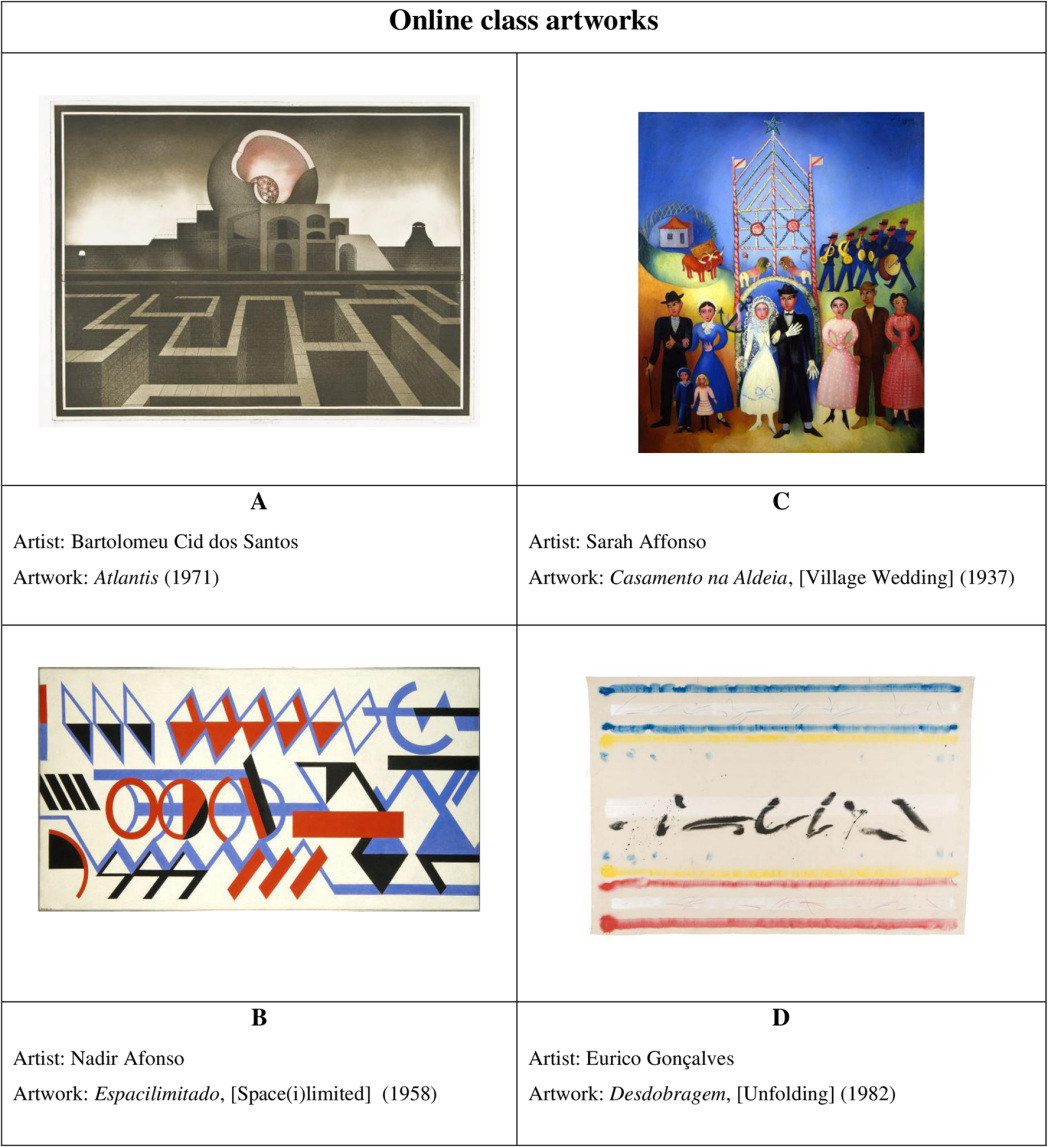
Selection and labelling of the 4 artworks presented at the online class.

After the intro clip, four different online class modules were randomly presented, pertaining to different works of art. According to the above, the chosen works are labelled A, B, C and D. The selection of artworks was made from the Fundação Calouste Gulbenkian publication named *Primeiro Olhar* (2002), an integrated Visual Arts Education Program^37^. The use of reproductions of these artworks is lawful, not having a lucrative purpose as they are intended for academic use only (Fig. 3).

### The Visual Analogue Scale (VAS)

Immediately after the video presentation, the participants should indicate on 2 different VAS scales the level of mental and physical fatigue perceived at that moment. Visual Analogue Scales are commonly used to measure magnitude of internal states such as pain stress, anxiety, mood and various functional capabilities^30,31^. VAS is a psychometrical measurement instrument that makes use of self-reported quantity of symptoms, emotional states, and attitudes. Its advantage as a measurement instrument relies in a format that covers a range of continuous values, for subjective indicators that cannot easily be directly measured. Since a VAS can measure any subjective construct, we decided to use it to measure the subjective feeling of physical fatigue and the subjective feeling of mental fatigue as the VAS is sensitive to small changes in intensity. The VAS is also very useful because the line bisection can then be converted into mm which translate into a numerical score that can be parametrically analysed^33^. The use of VAS with DHH populations has been documented as an instrument to determine degree and type of hearing loss and has proven to have an important role in measuring hard of hearing participants´ perceptions, especially with young adult populations^38^.

A VAS can be presented either vertically or horizontally. It takes the shape of a 100 mm line without numerical anchors. The participant is required to bisect the line more to the left or right (more to the top or to the bottom) depending on how much the subjective construct is felt. Thus, the VAS is useful in situations wherein a subjective construct needs to be measured without the bias of numerical anchors.

After submitting the form, a conversion to numerical values is generated, allowing for a parametric analysis of the results. In the first VAS the participants are prompted to answer the following question: “After completing this task I feel mentally…”. And in the second VAS the participants answer the following question: “After completing this task I feel physically…”.

### The performance test

Following the VAS completion, a set of ten online questions about the information conveyed were presented, using the multiple-choice modality for answering. This task did not impose a pre-set time limit, and the time taken to complete the performance test by each group was later analyzed. Questions such as: "A work painted on wet cloth was presented that represents a gestural attitude. Tick the false option." (a) The artist used a varied palette of colours, (b) The preparation of support takes longer than the process of painting; (c) The artist retouches the painting when the result does not match what he/she expected or (d) The work is carried out without intellectual content default”; “From the sequence of artists in the video, in what position was it presented the artist who had as a professor in college named Columbano Bordalo Pinheiro? Please select one of the given options”: (a) 1st position, (b) 2nd position, (c) 3rd position or (d) 4th position and “One of the works presented depicts a rural ceremony—a wedding. What figures are represented in the background (plane farthest from the observer)? Please select one of the given options”: (a) the guests; (b) the music band; (c) the bride and groom or (d) the house and the oxen. These answers provided a score of accurateness of the responses related to the visualized presentation.

## Data analysis

We used SPSS Statistics (IBM SPSS Statistics for Windows, Version 27.0) to compute Analyses of Variance (ANOVA) of the scores of the Fatigue Assessment Scale, of the Visual Analogue Scale and of the Performance test.

### Visual art literacy test

Normality and homogeneity between groups requirements were fulfilled. We computed a One-way ANOVA to investigate if the visual art literacy test scores differed between groups, *p* values were set at 0.05. There were no significant differences between the 3 groups in the test, (M_DHH_ = 71.70, SD = 16.55; M_PSL_ = 70.17 SD = 17.56; M_C_ = 74.94, SD = 16.95) F (2) = 0.347, *p* = 0.709, $\upeta _{p}^{2}$ = 0.023).

### Fatigue Assessment Scale

As a validated instrument, FAS results are coded and interpretated through given procedures^26–28^ within the total score ranges from 10 to 50. A total score of < 22 indicates no fatigue (or normal fatigue) and FAS scores between 22 and 50 indicate substantial fatigue. Also, two subgroups distinguish fatigue levels (scores 22–34) from extreme fatigue levels (scores ≥ 35). According to the FAS interpretation protocol, we analyzed the FAS results comparing answers between the 3 groups. Thus, by summing the scores on all 10 questions and calculating the group mean, results show a score of substantial fatigue (≥ 22) for the DHH (M = 22.76); and normal fatigue for the Control and PSL groups (M = 20.47 and M = 18.82, respectively).

**Figure 4:**
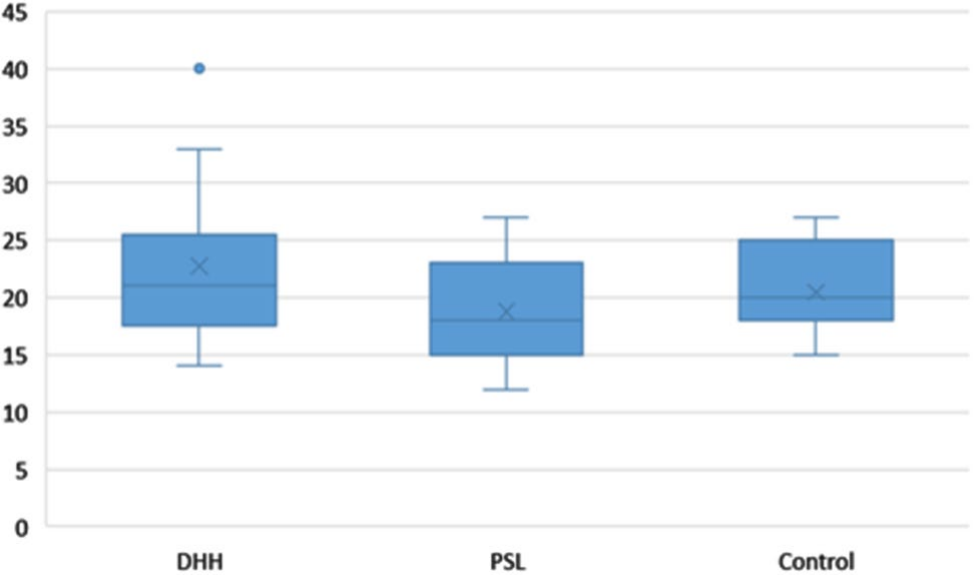
Boxplot of the FAS results with the minimum and maximum values, median, quartiles 1 and 3 and outlier value for the DHH.

For the FAS score dependent variable, the descriptive statistics for the DHH group are (Mean = 22.76; SD = 6.505) for the PSL (Mean = 18.82; SD = 4.334) and for the Control group (Mean = 22.47; SD = 3.875). ANOVA did not reveal significant differences between the 3 groups under analysis (F = 2.625, *p* = 0.083, Ƞ^2^ = 0.119). (Fig. 4).

### Visual Analogue Scale (VAS)

A Visual Analogue Scale (VAS) is a measurement instrument that indicates, from the participant’s perspective, their subjective perception of a certain characteristic across a continuum of a 100 mm line, in a vertical or horizontal position, anchored by numerical or word descriptors at each side.

**Figure 5:**
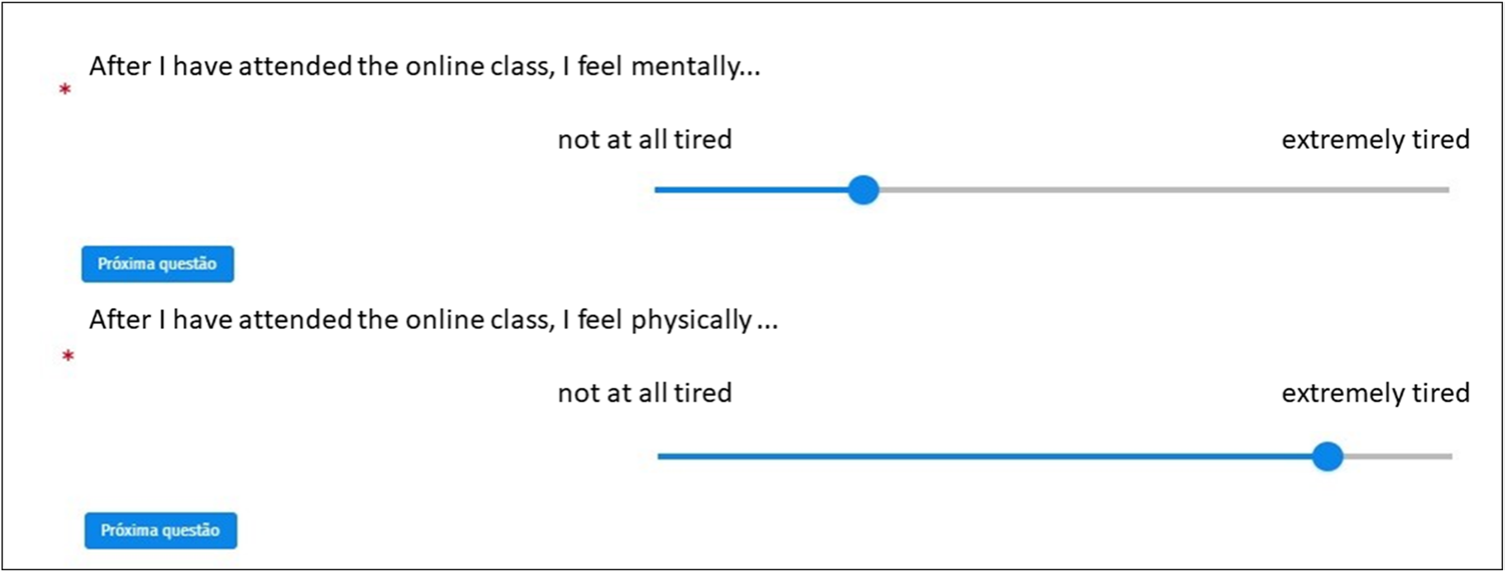
Caption from VAS slider bars (VAS_mental and VAS_physical). The points selected on the slider bars are random.

Here, the word descriptors were linked to fatigue levels, i.e., on the left side we indicated “not at all tired” and on the right side we indicated “extremely tired”. The scale was presented online with a digitally sensitive slider bar format. The participants were instructed to slide the bar between anchors to report their subjective feeling of fatigue. By dragging the slider bar, the participant indicated a higher value when approaching the right end (extremely tired), and a lower value or less fatigue, when sliding the bar towards the left end (not at all tired) (Fig. 5):

Although no values were presented, clicking on a point of the 100 mm line with the slider allowed for posterior conversion into a score between 0 and 100 for parametric analyses.

For both dependent variables VAS_Mental and VAS_Physical, statistical relevant differences are shown between groups.

For the VAS_Mental fatigue variable [(F(2) = 3.911, *p* = 0.027, $\upeta _{p}^{2}$ = 0.333], DHH present highest mean (M = 50.58, SD = 28.71), followed by PSL (M = 45.88, SD = 20.25) and Control group (M = 29.41, SD = 24.49). For VAS_Mental One-way ANOVA Post hoc Bonferroni correction shows that the DHH group significantly differs from the Control group (*p* = 0.032) and no significant differences were found between DHH and PSL (*p* = 1.000), or between PSL and the Control group (*p* = 0.131). A large size effect was verified by Cohen’s D (d = 0.82) in mental fatigue dimension between DHH and Control group.

**Figure 6:**
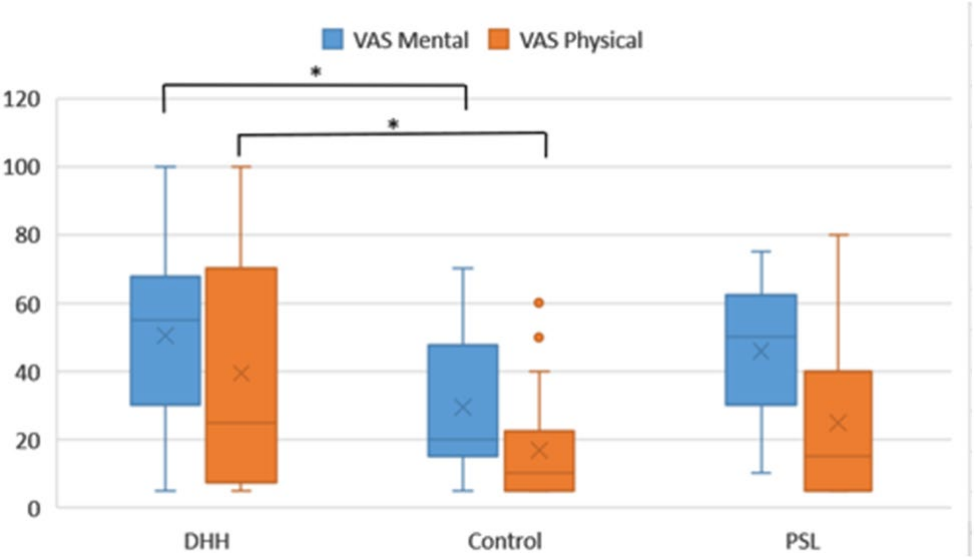
Boxplot with VAS_mental and VAS_Physical distributions with the minimum and maximum values, median, quartiles 1 and 3 and outlier values. Asterisks indicate significant comparisons (*p* < 0.05).

For the VAS_Physical fatigue variable [(F(2) = 3.245, *p* = 0.048, $\upeta _{p}^{2}$ = 0.119] the DHH group presents the highest mean (M = 39.41, SD = 33.86), followed by the PSL and Control groups (M = 25.00, SD = 24.81; M = 16.76, SD = 17.40, respectively). Bonferroni's post hoc test was used with adjustment for multiple comparisons, and a significant difference was found, for *p* < 0.05, between the DHH group and the Control group (*p* = 0.046). We calculated Cohen's D to verify the magnitude of the effect for this difference and observed a high magnitude of the effect (d = 0.87) (Fig. 6).

### The multiple-choice performance test

**Figure 7:**
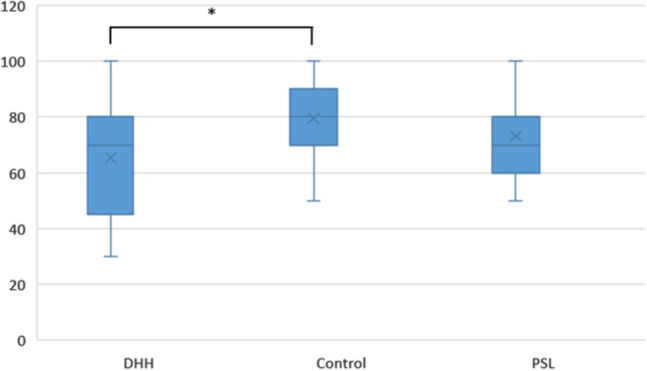
Boxplot of the Performance Test scores across groups, with the minimum and maximum values, median, quartiles 1 and 3. Asterisk indicate significant comparisons (*p* < 0.05).

As already mentioned, the online class purposely reproduced a traditional model, commonly used in academia, but not only. Meetings, conferences, or seminars share the same type of display, combining images, text, presenter/lecturer voice and sign language translation. This ecological situation delivered to participants as framed in our experimental design, followed the recognition of adverse effects that poor or inadequate multimedia instructional material brings to DHH students, since it is recognized in recent studies on the use of educational technologies, in distance learning for the deaf during the pandemic, that the presence of an interpreter on the screen might lead to erroneous assumptions on the accessibility and efficacy of online classes^22^. Accordingly, we wanted to know the extent of these adverse effects in the performance dimension, in which the participants had to recall the information received in the online class to respond to a set of ten questions. As we claimed before, our hypothesis is that the DHH performance would be below the other group results, so we computed a one-tailed analysis for statistical significance. One way ANOVA for correlated samples test shows differences between the 3 groups in the Performance total score [F(2) = 2.998; *p* = 0.024, Ƞ^2^ = 0.55] with the DHH (M = 65.29, SD = 21.82) having significantly lower mean in the Performance Test compared to the Control group (M = 79.41, SD = 13.90, yet not differing from the PSL group (M = 73.58, SD = 13.65) (Fig. 7). A high effect dimension was found by calculating Cohen’s D between the DHH and Control group (d = 0.80).

The time spent in completing the performance test was as follows for each of the groups (in minutes): M_DHH_ = 11.06; SD = 202.57, M_PSL_ = 10.47; SD = 264.31, M_C_ = 10.09; SD = 581.63. The ANOVA test showed no significant differences for completion time between groups [F(2) = 0.107, *p* = 0.889, Ƞ^2^ = 0.004].

## Discussion

Here, we aimed to investigate how an *e-learning* environment, such as an online class, might differently affect the participant groups in this study, with focus in fatigue assessment, and performance. We recruited participants from three distinct groups, DHH participants, hearing participants, and a control group. We applied different instruments at different timings. The FAS and a Visual art literacy test when recruiting participants and a VAS at a post-task moment. The Visual art literacy test was used to assess the knowledge and understanding of the language and codes of the visual arts, without the influence of the subsequent experimental procedures. The three groups did not present any differences in such knowledge so we could assume that the putative differences in the performance post-task test would be due to how they were able to acquire and process the information conveyed during the online class. The FAS was used to obtain an initial baseline of everyday life fatigue and concerning the FAS questionnaire results, the DHH group revealed subtle higher rates, but no statistical differences were found between the three groups. The use of PSL was an important factor in the selection of participants due to the multimodal and bilingual nature of the instructional material used in the online class. Here, this variable does not seem to impact DPSL in daily fatigue when compared to the DHH group. As we didn’t find statistically relevant differences between groups, our FAS results differ from previously described self-reported results by groups of hearing-impaired individuals, i.e., listeners with hearing loss that reported high levels of listening effort wherein the experimental design included the use of FAS^39^. In fact, previous literature states that, daily, DHH adults report higher fatigue rates in a consistent way, associated with sustained visual attention combined with listening effort to grasp environment information and respond to cognitive tasks^1–3,6,13^. It is possible that the tasks that lead to such fatigue are more strenuous (such as an online class) than the daily tasks experienced by our participants. Indeed, when attending to the VAS scores (mental and physical), the DHH have the highest post-task fatigue scores and significantly differ from the Control group. The DHH group have the closest maximum values between mental and physical fatigue, indicating the relationship between the two fatigue dimensions in a post-task moment involving cognitive demands.

Furthermore, our results reveal an association between post-task fatigue rates and lower performance scores for the DHH. Optimal methods and tools used in the classroom, to direct and maintain visual attention, can prevent DHH students from visual attention strays and keep the connection to the delivered information that, otherwise, becomes tenuous increasing potential mental fatigue^40^. Again, the differences are significantly bigger when compared with the non-PSL users hearing participants. Here, and diverging from the FAS results, the PSL variable seems to have contributed to an increase in both mental and physical fatigue of the hearing group. Previous literature shows that individuals have a limited processing capacity and must select pertinent information from the multitude of available sensory input. This limitation is evidenced in the attention processing mechanism such as divided attention as it relates to the optimal allocation of resources between different sets of input by splitting or rapid shifting of the attentional focus, given the inability to process stimuli in one or several sensory modalities in parallel^41^. This process becomes more difficult with the quantity and complexity of the component tasks, suggesting that dividing attention between simultaneous stimuli intensifies and recruits additional neurocognitive resources, and may lead to limitations on attention span and cognitive load management^17,20^. Also, bilingual bimodal individuals might have experienced here the processing of code-blending stimuli (speech and sign simultaneously) which is analogous to a cognitive demanding sociolinguistic code-switching in communication i.e., it is harder to suppress a second language when that second language uses a different modality^15,41^. PSL individuals might have tried to suppress PSL to pay attention to the oral language (or the opposite), trying a complete suppression of the non-selected language and thus experiencing higher levels of fatigue.

According to the literature, visuospatial attention is altered by early deafness but, interestingly, research about the gaming experience with DHH adults has proven that training visual peripherical responses in gaming (videogames) have an important role in the achievement of better visuospatial attention control, that is, the type of response to gaming challenges might contribute to minor potential visuospatial distractions. However, in our online class, which strongly differs from a traditional classroom context, we acknowledge the inherent problems of the distribution of visual attention, since all the information conveyed was relevant, contrary to the studied effect of the video game experience, which manages to train visuospatial attention by a combination of relevant-irrelevant visual stimuli using Flanker tasks^42^.

We consider that, in our research, the augmentation of the attention stray and split-attention effect occurred in tandem with poorly designed instructional/educational materials, namely the inadequate design of multimedia instructional resource^17–20^. In fact, this effect was confirmed by the VAS fatigue scores, as we consider having presented an ecological *e-learning* situation which hardly meets the needs of DHH students, due to its problematic simultaneous stimuli input, with no concern for interactivity situations between presenter and participant, pauses between contents, opportunities to evoke and consolidate information and diversity in the designed modality for content presentation (e.g., screen display elements).

Also emphasized before, test performance times were, on average, similar in the 3 groups. These data lead to an unavoidable analysis of the issue of the duration of assessment moments in classes with DHH students as, in this case, no time limit was imposed to complete the task, and an extension period would not have positively influenced test scores for the DHH: this group performed the worst of the 3 groups and presented the highest fatigue rates.

From our analysis, the consistency of results between DHH and PSL group also stands out: the levels of mental and physical fatigue in post-task effort relates with lower performance scores, i.e., PSL nonusers feel less fatigue and achieve better performance scores.

Interestingly, an innovative dimension of our study emphasizes the situation of the hearing participants PSL users, mostly working as Sign Language Interpreters. We showed that for slight non-significant lower levels of daily fatigue (FAS), similar fatigue levels are obtained at post-task, when compared to DHH, as well as lower performance scores in the performance test. It is possible that this group (PSL) might have felt a similar cognitive overload and a subsequent fatigue sensation due to the limitations of the divided attentional mechanisms. That is, as they are PSL users and fluent in the dimension of oral Portuguese, the integration of information through simultaneous multimodal channels made it difficult to grasp the contents of the online class.

It should be noted that the relationship between stress and burnout in Sign Language Interpreters has been established in the literature confirming burnout dimensions such as emotional exhaustion, depersonalization, and personal accomplishment^43^. Although interpreters work situation may vary (e.g., daily working hours, different schedules, working location/setting), research has looked closely to some occupational demands that suggest possible predictors of stress and burnout in educational interpreters, such as workload, responsibility, perceived control, and co-workers support, among others. Investigation shows that educational interpreters experience high work demands, which are congruent with our experiment results, as these 2 groups might have been impacted by levels of distractibility with subsequent out-turn on both fatigue and test performance scores.

Concomitantly, our results are consistent with the literature review regarding the risk of ineffectiveness of poor or inadequate multimedia resources for the DHH population^17^, ^20^. We have also confirmed the need, according to the available literature, of optimizing interactive cognitive tasks in multimedia instructional design, as they help in creating more flexible and engaging learning dynamics, different cognitive demands as well as the opportunity to control fatigue through breaks and recovery time^8,22–25^.

Overall, our results indicate levels of mental and physical fatigue consistent with research in the field of deafness and cognitive load^19,21,23,24^, and the consequent constraints on the maintenance of attentional mechanisms^10,12^ in demanding cognitive tasks. Together, our results seem to show that, when DHH are asked to visualize the multimedia stimulus in the format presented in our research, there is a combination of factors that negatively affect both the apprehension of the conveyed information and simultaneously lead to an increase in levels of mental and physical fatigue. In line with previous research, our study sheds light into the attentional split mechanism affecting hearing participants who use PSL (bimodal and bilingual) but that does not seem to interfere with PSL non-users, on post task fatigue rates or performance scores^41^.

Given the frequent exposure of learning situations in the *e-learning* modality during the last two years with periods of confinement due to pandemic for COVID-19, we are aligned with research in the field of communication technologies and multimedia instructional material for DHH students, that assert the need to reconsider the limitation imposed by the combination of audio/video channels as unquestionable assumptions on which multimedia design theories and principles are based^14,17,18^. Our findings state a clear association between cognitive load and low achievement i.e., whenever cognitive load increases, the apprehension and memorization of the conveyed concepts decreases. In our study, in addition to the mental dimension, CL is also self-reported in terms of physical fatigue. These results are confirmed by the principles of Cognitive Load theory and outline the importance of prioritizing the assumptions upon which Cognitive theory of multimedia learning stands on the design of educational materials that reduce CL, enhancing effective learning^17,20,21,24,25.^

## Conclusion

The *e-learning* reality is not an unprecedented reality, namely in the educational field. Given the great adhesion to information and communication technologies, the DHH population is generally elected for distance learning modalities. However, during the pandemic period triggered by Covid-19 and the post-pandemic transition, there was an exponential increase in this type of knowledge transmission, whose benefits, in certain situations, outweigh the losses of in-person training, class or lecture^22^. For the DHH population, these benefits are not evident, and here we demonstrate different levels of possible harm. Faced with higher fatigue rates and lower performances, the DHH population might be at disadvantage in the several dimensions of academic challenges, leading to further inequalities and constraints that affect well-being and participation opportunities. With this research we hope to contribute to the discussion concerning the pros and cons of digital migration and shed new light that might help redesign more accessible and equitable methodologies and approaches, especially in the DHH educational field, ultimately supporting policymakers in redefining optimal learning strategies.

## Supplementary Information

Supplementary Information 1.

## Data Availability

All relevant data are within the manuscript and its Supporting Information files.
