# Supplementary material for: E-learning is a burden for the deaf and hard of hearing
Source: Sci Rep. 2022 Jun 4;12:9346. doi: 10.1038/s41598-022-13542-1 (PMC9166930; doi:10.1038/s41598-022-13542-1)
Supplement: Supplementary file 1 — Supplementary Information 1. [file 41598_2022_13542_MOESM1_ESM.pdf]

Fatigue Assessment Scale (FAS)

Gender: ○ male ○ female Age (in years): [ ]

The following ten statements refer to how you usually feel. Per statement you can choose one out of five answer categories, varying from Never to Always. Please give an answer to each question, even if you do not have any complaints at the moment: Never, Sometimes (about monthly or less); Regularly (about a few times a month); Often (about weekly) and Always (about every day).

1. I am bothered by fatigue.

   ○ Never ○ Sometimes ○ Regularly ○ Often ○ Always

2. I get tired very quickly.

   ○ Never ○ Sometimes ○ Regularly ○ Often ○ Always

3. I don't do much during the day.

   ○ Never ○ Sometimes ○ Regularly ○ Often ○ Always

4. I have enough energy for everyday life.

   ○ Never ○ Sometimes ○ Regularly ○ Often ○ Always

5. Physically, I feel exhausted.

   ○ Never ○ Sometimes ○ Regularly ○ Often ○ Always

6. I have problems to start things.

   ○ Never ○ Sometimes ○ Regularly ○ Often ○ Always

7. I have problems to think clearly.

○ Never ○ Sometimes ○ Regularly ○ Often ○ Always

8. I feel no desire to do anything.

○ Never ○ Sometimes ○ Regularly ○ Often ○ Always

9. Mentally, I feel exhausted.

○ Never ○ Sometimes ○ Regularly ○ Often ○ Always

10. When I am doing something, I can concentrate quite well.

○ Never ○ Sometimes ○ Regularly ○ Often ○ Always

When all questions are answered, please push the button >> Ready

© ild care foundation; www.ildcare.nl; is only allowed with the permission of the ild care foundation: info@ildcare.nl.
Developed by Gosker Digital Solutions

Referenties:

Drent M, Lower EE, De Vries J. Sarcoidosis-associated fatigue. Eur Respir J 2012; 40: 255–263. http://www.ncbi.nlm.nih.gov/pubmed/22441750

Kleijn WPE, De Vries J, Wijnen PAHM, Drent M. Minimal (clinically) important differences for the Fatigue Assessment Scale in sarcoidosis. Respir Med 2011; 105: 1388-95. http://www.ncbi.nlm.nih.gov/pubmed/21700440

De Vries, Michielsen H, Van Heck GL, Drent M. Measuring fatigue in sarcoidosis: the Fatigue Assessment Scale (FAS). Br J Health Psychol 2004; 9: 279-91. http://www.ncbi.nlm.nih.gov/pubmed/15296678

ild care foundation
